# Role of Silicon on Plant–Pathogen Interactions

**DOI:** 10.3389/fpls.2017.00701

**Published:** 2017-05-05

**Authors:** Min Wang, Limin Gao, Suyue Dong, Yuming Sun, Qirong Shen, Shiwei Guo

**Affiliations:** Jiangsu Provincial Key Lab for Organic Solid Waste Utilization, National Engineering Research Center for Organic-Based Fertilizers, Jiangsu Collaborative Innovation Center for Solid Organic Waste Resource Utilization, Nanjing Agricultural UniversityNanjing, China

**Keywords:** silicon, plant–pathogen interactions, physical, biochemical, molecular, defense response

## Abstract

Although silicon (Si) is not recognized as an essential element for general higher plants, it has beneficial effects on the growth and production of a wide range of plant species. Si is known to effectively mitigate various environmental stresses and enhance plant resistance against both fungal and bacterial pathogens. In this review, the effects of Si on plant–pathogen interactions are analyzed, mainly on physical, biochemical, and molecular aspects. In most cases, the Si-induced biochemical/molecular resistance during plant–pathogen interactions were dominated as joint resistance, involving activating defense-related enzymes activates, stimulating antimicrobial compound production, regulating the complex network of signal pathways, and activating of the expression of defense-related genes. The most previous studies described an independent process, however, the whole plant resistances were rarely considered, especially the interaction of different process in higher plants. Si can act as a modulator influencing plant defense responses and interacting with key components of plant stress signaling systems leading to induced resistance. Priming of plant defense responses, alterations in phytohormone homeostasis, and networking by defense signaling components are all potential mechanisms involved in Si-triggered resistance responses. This review summarizes the roles of Si in plant–microbe interactions, evaluates the potential for improving plant resistance by modifying Si fertilizer inputs, and highlights future research concerning the role of Si in agriculture.

## Introduction

Silicon (Si) is the second most abundant element after oxygen in the earth’s crust, and comprises up to 70% of soil mass (Epstein, 1994; Savant et al., 1997; Ma and Yamaji, 2006). Si was initially not recognized as an essential element for higher plants, although it was known to be beneficial for plant growth and production. Its accumulation among plant species differs greatly, due to differences in root Si uptake capacity (Takahashi et al., 1990). Generally, Si uptake takes place through plant roots as silicic acid [Si(OH)_4_], an uncharged molecule (Ma and Yamaji, 2006), and passes through the plasma membrane via two Si transporters, Lsi1 and Lsi2, which function as influx transporters and efflux transporters, respectively (Ma et al., 2006, 2007, 2008).

Numerous studies show that Si accumulates in plants and exerts various beneficial effects for many plant species, especially gramineous plants such as rice and sugarcane and some cyperaceous plants (Epstein, 1994, 1999; Liang, 1999; Liang et al., 2005b). Absorbed Si is mainly deposited in cell walls, and is also involved with stress-related signaling systems (Fauteux et al., 2005). Si is helpful for improving the mechanical and physiological properties of plants and contributes to plants overcoming many biotic and abiotic stresses (Epstein, 1999; Richmond and Sussman, 2003; Ma, 2004; Ma and Yamaji, 2006). For example, Si enhances resistance to diseases caused by fungi, bacteria, and pests (Fauteux et al., 2005; Marschner, 2012), as well as exerting alleviative effects on various abiotic stresses including lodging, drought stress, salt stress, water logging, metal toxicity, nutrient imbalance, radiation damage, high temperature, freezing, and UV in a wide variety of plant species (Epstein, 1994, 1999; Savant et al., 1997; Ma, 2004; Ma and Yamaji, 2006; Liu et al., 2014; Coskun et al., 2016).

Many studies have focused on the role of Si on plant–microbe interactions and enhanced host resistance to a range of microbial pathogens by stimulating defense reactions (Remus-Borel et al., 2005; Cai et al., 2008; Ghareeb et al., 2011; Ye et al., 2013). However, the mechanistic basis and regulation of Si-mediated disease resistance are still poorly understood. Furthermore, the underlying mechanisms of Si regulated plant–microbe interactions have not been identified so far in higher plants. In this review, the effect of Si on plant–microbe interactions are discussed, and the physical, biochemical, and molecular regulatory mechanisms of Si on plant disease resistance are extensively reviewed.

Plant diseases are a major threat to agricultural production as they cause serious loss of crop yield and quality. Numerous studies have reported that Si is effective in controlling diseases caused by both fungal and bacterial pathogens in different plant species (Fauteux et al., 2005; Rodrigues and Datnof, 2015). A priming role of Si has been demonstrated in plant-pathogen interactions and the regulation of Si in plant diseases is summarized in **Table 1**. Si plays a positive role in plant–pathogen interactions and increases plant resistance to disease caused by fungi, bacteria, viruses, and nematodes.

**Table 1:** Effects of silicon on plant disease and related resistance mechanisms.

Hosts	Diseases	Pathogens	Effects	Reference	Resistance mechanisms
*Arabidopsis*	Powdery mildew	*Erysiphe cichoracearum, Agrobacterium tumefaciens*	+	Ghanmi et al., 2004; Fauteux et al., 2006; Vivancos et al., 2015	Physical, biochemical and molecular
Banana	Black sigatoka	*Mycosphaerella fijiensis*	+	Kablan et al., 2012	Physical and biochemical
	Fusarium wilt	*Fusarium oxysporum* f. sp. *cubense*	+	Fortunato et al., 2012a	Physical and biochemical
	Root rot	*Cylindrocladium spathiphylli*	+	Vermeire et al., 2011	Biochemical
	Xanthomonas wilt	*Xanthomonas campestris*	+	Mburu et al., 2015	Physical and biochemical
Barley	Powdery mildew	*Blumeria graminis*	+	Wiese et al., 2005	Physical
Bean	Angular leaf spot	*Pseudocercospora griseola*	+	Rodrigues et al., 2010	Physical
Belle pepper	Phytophthora blight	*Phytophthora capsici*	+	French-Monar et al., 2010	Physical
Bentgrass	Dollar spot	*Sclerotinia homoeocarpa*	+	Uriarte et al., 2004; Zhang et al., 2006	Physical and biochemical ?
Bitter gourd	Powdery mildew	*Erysiphe* sp.	+	Ratnayake et al., 2016	Biochemical
Capsicum	Anthracnose	*Colletotrichum gloeosporioides*	+	Jayawardana et al., 2016	Physical and biochemical
Cherry	Fruit decay	*Penicillium expansum, Monilinia fructicola*	+	Qin and Tian, 2005	Biochemical
Chinese cantaloupe	Fusarium root rot	*Fusarium* spp.	+	Liu et al., 2009	Physical and biochemical
	Postharvest pink rot	*Trichothecium roseum*	+	Guo et al., 2007	Physical and biochemical
Coffee	Leaf rust	*Hemileia vastatrix*	+	Carré-Missio et al., 2014	Physical
	Root-knot Nematode	*Meloidogyne exigua*	+	Silva R. et al., 2010	Biochemical
Common bean	Anthracnose	*Colletotrichum lindemuthianum*	+	Polanco et al., 2014; Rodrigues et al., 2015a	Biochemical
Cotton	Fusarium wilt	*Fusarium oxysporum* f. sp. *vasinfectum*	+	Whan et al., 2016	Physical and biochemical
Creeping, turf grass	Brown patch	*Rhizoctonia solani*	+	Uriarte et al., 2004; Zhang et al., 2006	Physical and biochemical?
Cucumber	Crown and root rot	*Pythium ultimum*	+	Chérif et al., 1994	Biochemical
	Fusarium wilt	*Fusarium oxysporum* f. sp. *cucumerinum*	+	Miyake and Takahashi, 1983	Physical and biochemical?
	Powdery mildew	*Sphaerotheca fuliginea, Podosphaera xanthii*	+	Menzies et al., 1991, 1992; Fawe et al., 1998; Liang et al., 2005a	Physical and biochemical
Gerbera daisy	Powdery mildew	*Erysiphe cichoracearum, Podosphaera fusca*	/	Moyer et al., 2008	/
Hami melons	Decay	*Alternaria alternate, Fusarium semitectum, Trichothecium roseum*	+	Bi et al., 2006	Biochemical
Lettuce	Downy mildew	*Bremia lactucae*	+	Garibaldi et al., 2011	Physical and biochemical?
Melon	Bacterial fruit blotch	*Acidovorax citrulli*	+	Conceição et al., 2014	Biochemical
	Powdery mildew	*Podosphaera xanthii*	+	Dallagnol et al., 2015	Biochemical
Muskmelon	Pink rot disease	*Trichothecium roseum*	+	Li et al., 2011	Biochemical
	Powdery mildew	*Sphaerotheca fuliginea*	+	Menzies et al., 1992	Physical and biochemical
Oil palm	Basal stem rot	*Ganoderma boninense*	+	Najihah et al., 2015	Physical
Pea	Brown spot	*Mycosphaerella pinodes*	+	Dann and Muir, 2002	Biochemical
Pearl millet	Downy mildew	*Sclerospora graminicola*	+	Deepak et al., 2008	Physical and biochemical
Perennial ryegrass	Fusarium patch	*Microdochium nivale*	+	McDonagh and Hunter, 2010	Physical
	Gray leaf spot	*Magnaporthe oryzae*	+	Rahman et al., 2015	Biochemical
Potato	Dry rot	*Fusarium sulphureum*	+	Li et al., 2009	Biochemical
Pumpkin	Powdery mildew	*Podosphaera xanthii*	+	Lepolu Torlon et al., 2016	Physical and biochemical?
Rice	Blast	*Pyricularia oryzae, Magnaporthe grisea, Magnaporthe oryzae*	+	Seebold et al., 2000; Kim et al., 2002; Rodrigues et al., 2003; Cai et al., 2008, Hayasaka et al., 2008; Brunings et al., 2009; Domiciano et al., 2015	Physical, biochemical and molecular
	Brown spot	*Bipolaris oryzae, Cochliobolus miyabeanus*	+	Dallagnol et al., 2011, 2013; Prabhu et al., 2012; Van et al., 2015a	Physical, biochemical and molecular
	Grain discoloration	*Bipolaris oryzae*	+	Prabhu et al., 2012	Molecular
	Leaf scald	*Monographella albescens, Microdochium oryzae*	+	Tatagiba et al., 2016; Araujo et al., 2015	Physical and biochemical
	Sheath blight	*Rhizoctonia solani*	+	Peters et al., 2001; Schurt et al., 2014	Physical and biochemical
Rose	Powdery mildew	*Podosphaera pannosa*	+	Shetty et al., 2012	Physical
Sorghum	Anthracnose	*Colletotrichum sublineolum*	+	Resende et al., 2013	Physical and biochemical ?
Soybean	Phytophthora stem and root rot	*Phytophthora sojae*	+	Guérin et al., 2014	Molecular
	Rust	*Phakopsora pachyrhizi*	+	Cruz et al., 2014; Lemes et al., 2011	Biochemical
St. Augustinegrass	Gray leaf spot	*Magnaporthe grisea*	+	Brecht et al., 2007	Physical and biochemical
Strawberry	Powdery mildew	*Sphaerotheca aphanis*	+	Kanto et al., 2006	Physical and biochemical
Sugarcane	Brown rust	*Puccinia melanocephala*	+	Ramouthar et al., 2015	Physical and biochemical ?
Tall fescue	Brown patch	*Rhizoctonia solani*	-	Zhang et al., 2006	/
Tobacco	Viral infection	*Tobacco ringspot virus*	+	Zellner et al., 2011	Molecular
		*Tobacco mosaic virus*	/	Zellner et al., 2011	/
Tomato	Bacterial speck	*Pseudomonas syringae*	+	Andrade et al., 2013	Biochemical
	Bacterial wilt	*Ralstonia solanacearum*	+	Ghareeb et al., 2011; Chen et al., 2014	Molecular
	Fusarium crown and root rot	*Fusarium oxysporum* f. sp *radicis-lycopersici*	+	Huang et al., 2011	Physical
Tomato, bitter gourd	Root rot	*Pythium aphanidermatum*	+	Heine et al., 2007	Biochemical and molecular?
Wheat	Blast	*Pyricularia grisea*	+	Filha et al., 2011	Physical and biochemical
	Leaf blast	*Pyricularia oryzae*	+	Silva et al., 2015	Biochemical
	Leaf streak	*Xanthomonas translucens*	+	Silva I.T. et al., 2010	Physical and biochemical
	Powdery mildew	*Blumeria graminis*	+	Chain et al., 2009; Guével et al., 2007; Moldes et al., 2016	Physical, biochemical and molecular
	Spot blotch	*Bipolaris sorokiniana*	+	Domiciano et al., 2010	Physical and biochemical
Zucchini squash	Powdery mildew	*Erysiphe cichoracearum, Podosphaera xanthii*	+	Menzies et al., 1992; Savvas et al., 2009	Physical and biochemical

*Positive (+), negative (-) or no effect (/) of silicon on plant resistance to disease. ?, indicates possible defense mechanisms are involved.*

Silicon could alleviate plant disease through preventing pathogen penetration (1) via structural reinforcement (Epstein, 1999; Epstein, 2001; Rodrigues et al., 2015b), (2) by inhibiting pathogen colonization through stimulating systemic acquired resistance, (3) through antimicrobial compound production (Fauteux et al., 2005; Datnoff et al., 2007; Fortunato et al., 2012b; Van et al., 2013), as well as (4) through increasing plant resistance by activating multiple signaling pathways and defense-related gene expression (Fauteux et al., 2005; Chen et al., 2014; Vivancos et al., 2015). The beneficial effects of Si with regard to plant resistance to disease are attributed to Si accumulation in epidermal tissue, the formation of complexes with organic compounds in cell walls, the induction of phenolic compounds, phytolexin/glucanase/peroxidase production, and regulating pathogenicity or stress-related gene expression to limit pathogen invasion and colonization (Belanger et al., 2003; Brunings et al., 2009; Chain et al., 2009; Sakr, 2016). The effect of Si on plant–microbe interactions and related physical, biochemical, and molecular resistance mechanisms have been demonstrated in **Table 1** and will be detailed discussed in the following section.

## Silicon-Mediated Disease Resistance

### Physical Mechanisms

The beneficial effects of Si on plant growth are attributed to improved overall mechanical strength and an outer protective layer (Epstein, 1999, 2001; Sun et al., 2010). Successful infection requires plant pathogens to enter the host plant by penetrating physical barriers including wax, cuticles, and cell walls (Schmelzer, 2002; Nawrath, 2006; Łaźniewska et al., 2012).

Silicon-enhanced resistance is associated with the density of silicified long and short epidermal cells, the thick layer of silica under the cuticle, the double cuticular layer, the thickened Si-cellulose membrane, formation of papilla, and complexes formed with organic compounds in epidermal cell walls that strengthen plants mechanically. The physical barriers inhibit pathogen penetration and make plant cells less susceptible to enzymatic degradation caused by fungal pathogen invasion (Inanaga et al., 1995; Fauteux et al., 2005; Datnoff et al., 2007; Van et al., 2013).

**FIGURE 1:**
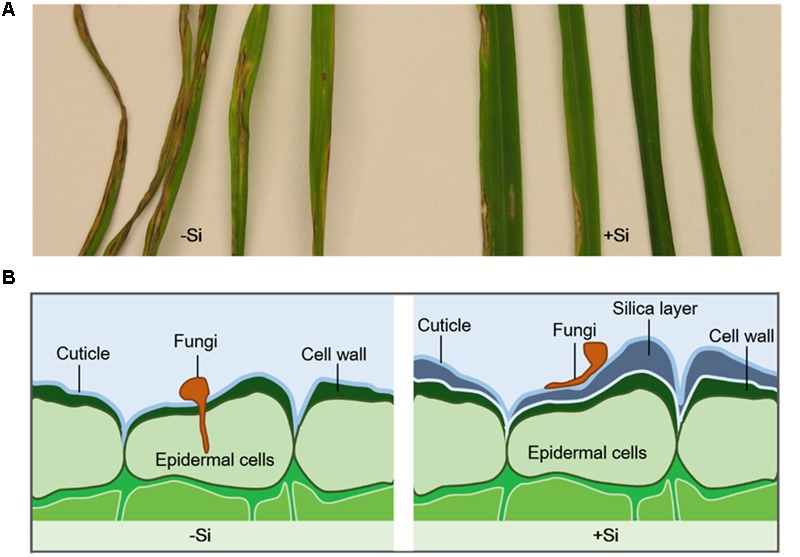
**(A)** Leaf blast symptoms in rice after inoculated with *Magnaporthe grisea* for 10 days (Sun et al., 2010). Rice plants were continuously treated with (+Si) or without silicon (–Si). **(B)** Silica layer was formed in the cell wall of Si-treated plants and enhanced plant resistance to fungi infection by physical barriers.

Silicon accumulates and, when deposited beneath the cuticle, can form a cuticle-Si double layer to prevent pathogen penetration, thereby decreasing disease incidence (**Figure 1**) (Ma and Yamaji, 2006, 2008). Most Si is cross-linked with hemicellulose in cell walls, which improves mechanical properties and regeneration (He et al., 2015; Guerriero et al., 2016). Si contributes not only to cell-wall rigidity and reinforcement, it also increases cell-wall elasticity during extension growth (Marschner, 2012). In primary cell walls, Si interacts with cell-wall constituents such as pectins and polyphenols, which increase cell-wall elasticity during extension growth (Emadian and Newton, 1989). In rice, Si-induced epidermal cell-wall fortification is associated with reduced severity of blast disease (Kim et al., 2002). Si application restricted hyphael entry to the first-invaded epidermal cell for wheat leaves infected with *Pyricularia oryzae*, while hyphae successfully invaded several neighboring leaf cells when there was no Si treatment (Sousa et al., 2013). A similar result was found in wheat (*Bipolaris sorokiniana*) pathosystem (Domiciano et al., 2013), in which Si supply delayed pathogen ingress into epidermal cells and reduced fungal colonization in foliar tissue. For rice infected with *Pyricularia grisea* and *Rhizoctonia solani*, a decrease in the number of leaf blade lesions was associated with an increased incubation period when Si was deposited on tissue surfaces (Rodrigues et al., 2001; Seebold et al., 2004). Moreover, the number of successful penetrative appressorial sites for *P. oryzae* was decreased in rice supplied with Si, suggesting that the denser Si layer contributed to preventing or delaying pathogen penetration (Hayasaka et al., 2008).

Besides the reinforcement of cell walls by Si, the formation of papillae has also been stimulated by Si during pathogen infection. Silicon accumulation was found to occur in the haustorial neck and collar area of fungus as well as in papillae, which contributed to preventing pathogen invasion (Samuels et al., 1994). Zeyen et al. (1993) demonstrated that barley epidermal cells could produce papillae in response to *Blumeria graminis* f. sp. *hordei* infection during Si application. A similar result has been found in the rose, in which Si supply increased the number of papillae in leaf cells in response to *Podosphaera pannosa* infection (Shetty et al., 2012). The prevalence of papillae after Si treatment could increase rice resistance to blast (Cai et al., 2008), wheat and barley resistance to powdery mildew (Zeyen et al., 1993; Belanger et al., 2003).

Heine et al. (2007) reported that the ability of Si to inhibit fungal spread in root apices is dependent on the uptake of Si into root symplasts. Further, the accumulation of Si on root cell walls did not represent a physical barrier to the spread of *Pythium aphanidermatum* in tomato or bitter gourd roots. In cucumber plants, Si foliar application could increase cucumber resistance to powdery mildew via physical barrier and osmotic effects, but Si root application can induce systemic resistance (Liang et al., 2005a). Taken together, Si, which is deposited in the wax, cuticle, and cell wall, as well as papillae, contributes in part to increased physical resistance against pathogen penetration. However, it is suggested that biochemical resistance to pathogens, as regulated by Si, is more complex than physical resistance alone; this has been strongly contested in recent years.

### Biochemical Mechanisms

Silicon-enhanced biochemical resistance is associated with (1) increasing the activity of defense-related enzymes, such as polyphenoloxidase, glucanase, peroxidase, and phenylalanine ammonia-lyase (PAL); (2) inducing antimicrobial compounds production, such as phenolic, flavonoids, phytoalexins and pathogenesis-related (PR) proteins in plants; and (3) regulating systemic signals, such as salicylic acid (SA), jasmonic acid (JA), and ethylene (ET; Fauteux et al., 2005; Datnoff et al., 2007; Fortunato et al., 2012b; Van et al., 2013).

#### Defense-Related Enzymes and Antimicrobial Compounds

Defense-related enzymes are closely linked with disease resistance, and Si has been reported to stimulate the activity of these enzymes during plant–pathogen interactions (Fauteux et al., 2005; Datnoff et al., 2007; Van et al., 2013). Several studies have reported the role of Si in disease resistance by activating defense-related enzyme activities such as chitinase, peroxidases, polyphenoloxidases, β-1,3-glucanase, phenylalanine ammonia-lyase, uperoxide dismutase, ascorbate peroxidase, glutathione reductase, catalase, lipoxygenase, and glucanase. PAL, involved in the synthesis of plant secondary antimicrobial substances, is essential for plant disease resistance responses (Waewthongrak et al., 2015). The higher PAL activity after Si treatment contributes to an accumulation of total soluble phenolic and lignin-thioglycolic acid derivatives in the leaves of banana and coffee plants, and this corresponds with low disease incidence (Silva R. et al., 2010; Fortunato et al., 2012b). Polyphenol oxidase (PPO), which mainly exists in cytoplasm in a free form or bound in chloroplasts, mitochondria, and other subcellular organelles, is the main enzyme of phenolic substance oxidation (Quarta et al., 2013); its activity has been positively correlated with plant disease resistance (Piperno, 2006). Furthermore, PPO was found to be involved in the synthesis of lignin and to increase the antibacterial ability of host plants (Song et al., 2016). Si application could also increase peroxidase (POD) and chitinase (CHT) activities, which play important roles in host–pathogen interactions. POD is involved in cell-wall reinforcement and the final steps of lignin biosynthesis, as well as the cross-linking of cell-wall proteins (Brisson et al., 1994), while CHT is one of the PR proteins that contribute to hydrolyze the cell walls of many phytopathogenic fungi (Pan and Ye, 1992; Shewry and Lucas, 1997).

Defense-related enzyme activities induced by Si may regulate gene expression related to enzyme synthesis; for example, the expression of genes encoding phenylalanine ammonia-lyase (*PALa* and *PALb*) and lipoxygenase (*LOXa*) were significantly up-regulated in Si-treated perennial ryegrass plants, associated with suppression of gray leaf spot (Rahman et al., 2015). Si could elevate the activities of defense-related enzymes (e.g., peroxidase and polyphenol oxidase) via enhancing or priming JA-inducible responses to herbivory in rice (Ye et al., 2013). The beneficial effects of Si for suppressing pathogen infections via an increase in the activities of defense-related enzymes have been found in the pathosystems of cucumber (*Pythium* spp. and *Podosphaera xanthii*), pea (*Mycosphaerella pinodes*), wheat (*Pyricularia oryzae*), rice (*Magnaporthe oryzae, Bipolaris oryzae, Rhizoctonia solani*, and *Pyricularia oryzae*), melon (*Trichothecium roseum* and *Podosphaera xanthii*), Chinese cantaloupe (*Trichothecium roseum*), bean (*Colletotrichum lindemuthianum*), perennial ryegrass (*Magnaporthe oryzae*), and soybean (*Corynespora cassiicola*; **Table 2**).

**Table 2:** Defense-related enzymes regulated by silicon in plant–pathogen interactions.

Hosts	Diseases	Pathogen	Defense-related enzymes	Reference
Bean	Anthracnose	*Colletotrichum lindemuthianum*	Superoxide dismutase, ascorbate peroxidase, glutathione reductase	Polanco et al., 2014
Cucumber	Crown and root rot	*Pythium* spp.	Chitinase, peroxidases, polyphenoloxidases	Chérif et al., 1994
	Powdery mildew	*Podosphaera xanthii*	Peroxidases, polyphenoloxidases, chitinases	Liang et al., 2005a
Melon	Pink rot	*Trichothecium roseum*	Peroxidase	Bi et al., 2006
	Powdery mildew	*Podosphaera xanthii*	Chitinases, superoxide dismutase, β-1,3-glucanase	Dallagnol et al., 2015
Chinese cantaloupe	Pink rot	*Trichothecium roseum*	Peroxidases, phenylalanine ammonia-lyase	Guo et al., 2007
Pea	Leaf spot	*Mycosphaerella pinodes*	Chitinase, β-1,3-glucanase	Dann and Muir, 2002
Perennial ryegrass	Gray leaf spot	*Magnaporthe oryzae*	Peroxidase, polyphenol oxidase	Rahman et al., 2015
Rice	Blast	*Magnaporthe oryzae, Pyricularia oryzae*	Glucanase, peroxidase, polyphenol oxidase, phenylalanine ammonia-lyase, superoxide dismutase, catalase, ascorbate peroxidase, glutathione reductase, lipoxygenase	Rodrigues et al., 2003, 2004, 2005; Cai et al., 2008; Domiciano et al., 2015
	Brown spot	*Bipolaris oryzae*	Chitinase, peroxidase	Dallagnol et al., 2011
	Sheath blight	*Rhizoctonia solani*	Phenylalanine ammonia-lyases, peroxidases, polyphenoloxidases, chitinases	Schurt et al., 2014
Soybean	Target spot	*Corynespora cassiicola*	Chitinases, β-1-3-glucanases, phenylalanine ammonia-lyases, peroxidases, polyphenol oxidases	Fortunato et al., 2015
Wheat	Blast	*Pyricularia oryzae*	Chitinases, peroxidases	Filha et al., 2011

A substantial response to defense-related enzymes is the change in antimicrobial substances; generally, lower disease incidence in plants after Si application are associated with a higher activity of defense-related enzymes, which induce the production and accumulation of antimicrobial compounds, such as phenols, flavonoids, phytoalexins, and PR proteins in plants after pathogen penetration (Chérif et al., 1994; Fawe et al., 1998; Rodrigues et al., 2004; Remus-Borel et al., 2005). However, the opposite effect was found in soybeans, in which Si application reduced the basal antioxidant enzyme activity of leaves during *Cercospora sojina* infection, leading to an increase in host susceptibility to frogeye leaf spot. These findings suggest that Si-induced resistance to plant disease was most likely due to the less than optimal conditioning of the antioxidant system (Telles Nascimento et al., 2016).

Antimicrobial compounds help higher plants to combat disease (Fauteux et al., 2005; Datnoff et al., 2007; Van et al., 2013), and Si has been documented to stimulate the accumulation of antimicrobial compounds, such as phenols, flavonoids, and phytoalexins during pathogen infection (Chérif et al., 1994; Fawe et al., 1998; Rodrigues et al., 2004; Remus-Borel et al., 2005); this may therefore contribute to the enhancement of defense-related enzyme activities. Defense-related antimicrobial phenols or lignin-associated polyphenolic compounds increased by Si resulted from the inducing activities of PAL and PPO following pathogen invasion (Rahman et al., 2015). Si-enhanced lignin and flavonoid production is attributed to higher PAL activity induced by Si; PAL converts *L*-phenylalanine into *trans*-cinnamic acid, which in turn is the precursor of lignin and flavonoids (Dixon et al., 2002; Hao et al., 2011).

Lignin and phenolic secondary metabolism play important roles in plant disease resistance. Si is involved in phenolic metabolism and lignin biosynthesis in plant cell walls (Marschner, 2012). It also increases lignin-carbohydrate complexes and lignin content in the epidermal cell wall of rice, and enhances plant resistance to blast disease (Inanaga et al., 1995; Cai et al., 2008). Si supply could increase the total concentration of soluble phenolic compounds in host plants and enhance plant disease resistance through delaying the growth of invading pathogens (Dallagnol et al., 2011; Fortunato et al., 2015). Flavonoids, another phenolic compound, are also induced by Si and enhanced rose plant resistance to *Podosphaera pannosa* (Shetty et al., 2012), and wheat resistance to *Pyricularia oryzae* (Silva et al., 2015).

Higher accumulation of phenolic and lignin or lignin-thioglycolic acid derivatives, due to Si treatment, fortified cucumber plants against damping-off (*Pythium ultimum*) (Chérif et al., 1994), wheat against powdery mildew (*Blumeria graminis*) (Belanger et al., 2003) and blast (*Pyricularia oryzae*) (Filha et al., 2011), *Arabidopsis* against powdery mildew (*Erysiphe cichoracearum*) (Ghanmi et al., 2004), soybean against target spot (*Corynespora cassiicola*) (Fortunato et al., 2015), melon against powdery mildew (*Podosphaera xanthii*) (Dallagnol et al., 2015), rice against blast disease (*Magnaporthe grisea*) (Cai et al., 2008), brown spot (*Bipolaris oryzae*) (Dallagnol et al., 2011), and sheath blight (*Rhizoctonia solani*) (Zhang et al., 2013).

Phytoalexins is recognized to be critical in plant defense against pathogen infection. Enhanced production of phytoalexins reduces the incidence of powdery mildew caused by *Podosphaera xanthii* in cucumber plants (Fawe et al., 1998), as well as blast caused by *M. grisea* in rice (Rodrigues et al., 2004, 2005). Si supply is reported to increase accumulation of the flavonoid phytoalexins in cucumber plants during *Podosphaera xanthii* infection (Fawe et al., 1998). Similar results have been found in rice, in which Si increased resistance to blast by stimulating the production of phytoalexins, such as momilactones A and B (Rodrigues et al., 2004, 2005). With regard to perennial ryegrass (*Magnaporthe oryzae*) pathosystems, Si-induced enhancement of phenolic acids, including chlorogenic acid and flavonoids, and relative levels of genes encoding PAL and lipoxygenase contributed to improved resistance to gray leaf spot disease (Rahman et al., 2015).

#### Systemic Signals

To prevent pathogen infection, host plants have developed a complicated immune system providing several layers of constitutive and inducible defense mechanisms, which are regulated by a complex network of signal transduction pathways (Grant et al., 2013). SA, JA, and ET play key roles in plant immunity networks and regulate plant defense responses (Clarke et al., 2000; Devadas et al., 2002). SA is mainly active against biotrophic and hemibiotrophic pathogens, whereas JA and ET are predominantly involved against necrotrophic pathogens (Pieterse et al., 2012).

Several studies have suggested that Si may regulate plant stress responses by modulating phytohormone homeostasis and signaling pathways (Zhang et al., 2004; Fauteux et al., 2006; Iwai et al., 2006; De Vleesschauwer et al., 2008; Brunings et al., 2009; Chen et al., 2009; Ghareeb et al., 2011; Reynolds et al., 2016). Plant phytohormones accumulate in Si-treated plants in response to pathogen invasion, wounding, or herbivory (Fauteux et al., 2006; Ye et al., 2013; Kim et al., 2014); for example, Si-induced rice defense against insect herbivores through JA accumulation (Ye et al., 2013) and regulated wound-induced JA biosynthesis (Kim et al., 2014). In Si-treated *Arabidopsis* plants infected with powdery mildew pathogen (*Erysiphe cichoracearum*), the biosynthesis of SA, JA, and ET in leaves was stimulated, leading to increased resistance (Fauteux et al., 2006). Similarly, tomato infected with *Ralstonia solanacearum* showed that Si triggers activation of the JA and ET signaling pathways (Zhang et al., 2004; Chen et al., 2009; Ghareeb et al., 2011). The stimulating effects of Si on the JA and ET signaling pathways in rice challenged with *Magnaporthe oryzae* demonstrate that the Si-mediated signaling pathway is critical for enhancing rice resistance to blast disease (Iwai et al., 2006; De Vleesschauwer et al., 2008; Brunings et al., 2009). However, Van et al. (2015a) suggest that Si-induced rice resistance to *Cochliobolus miyabeanus* is regulated independently of the classic hormones SA and JA, but that it does interfere with the synthesis and/or action of fungal ET. In the defense of *Arabidopsis* against powdery mildew, although Si increases the expression of genes encoding enzymes involved in the SA pathway, resistant phenotypes show a significantly decreased production of SA and expression of defense genes compared with susceptible controls, implying that Si-mediated resistance involves mechanisms other than SA-dependent defense responses (Vivancos et al., 2015).

**FIGURE 2:**
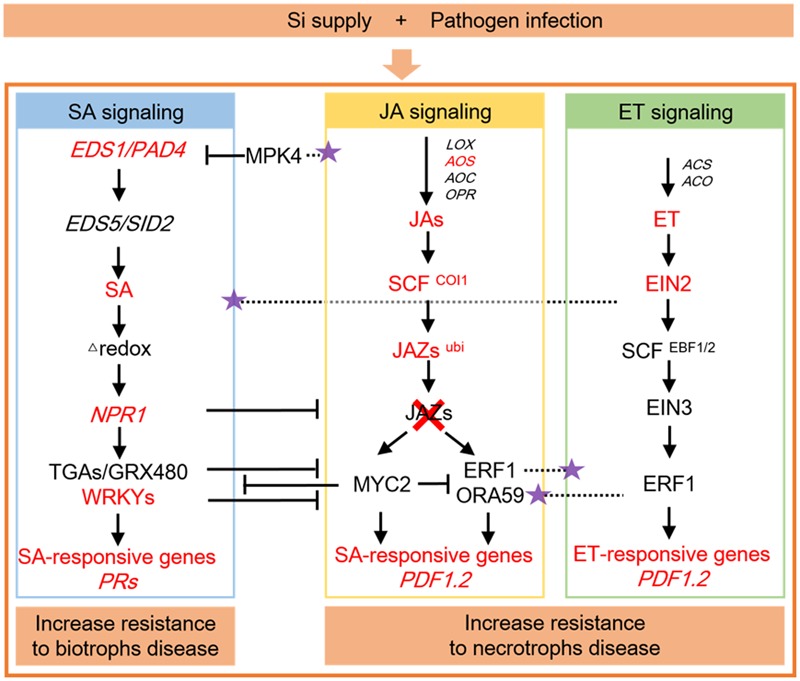
**Signaling pathways in the plant defense response regulated by silicon (Si).** Crosstalk between signaling pathways in plant defense originating from the actions of salicylic acid (SA), jasmonic acid (JA), and ethylene (ET) are demonstrated, as well as their interactions in modulating the defensive response regulated by Si. The SA signaling pathway mainly involves in biotrophs disease, whereas JA and ET signaling pathways attribute to necrotrophs disease. T, negative effect; purple stars, positive effect; red, increased or up-regulated by Si supply. The networking of signaling pathways are modified from Pieterse et al. (2009).

The signaling pathways in the plant defense response regulated by Si were demonstrated in **Figure 2**. The *EDS1* and *PAD4* genes are required for SA biosynthesis, whereas the *EDS5* and *SID2* genes involve in regulating SA biosynthesis (Shah, 2003). In *Arabidopsis*, the *TaLsi* plant, which contained higher Si, were more resistance to *Golovinomyces cichoracearum* infection than control plants when treated with Si, and corresponded with higher expressions of *EDS1* and *PAD4* genes, as well as *NPR1* and three SA-induced *PR* defense genes *PR1, PR2*, and *PR5* (Vivancos et al., 2015). Moreover, the mutants of *TaLsi1 sid2* and *TaLsi1 pad4*, which crossed mutants *pad4* and *sid2* with the line *TaLsi1*, showed lower area under the disease progress curve (AUDPC) after Si supply, suggesting that Si-enhanced resistance to *Golovinomyces cichoracearum* infection in *Arabidopsis* is maintained in *pad4* and *sid2* mutants engineered to better absorb Si (Vivancos et al., 2015). The regulatory protein NPR1 is critical for activation of *PR* gene expression in response to SA, and *NPR1* itself is positively regulated by some SA-inducible WRKY proteins (Li et al., 2004). During tomato plant infected with *R. solanacearum*, the gene expression of transcription factor *WRKY1* was upregulated in response to Si (Ghareeb et al., 2011). Si induced defense related genes and transcripts belong to the SA dependent pathway, which accompanied by an increase in the level of endogenous SA and subsequent *PRs* expression (Durrant and Dong, 2004; Kurabachew et al., 2013).

Silicon can induce expression of a large spectrum of inducible defense responses and amplifies the JA-mediated induced defense response by serving as a priming agent for the JA pathway (**Figure 2**), for example, the enhanced induction of defense-related enzymes and proteins, as well as enhanced induction of transcripts encoding proteins involved in JA signaling, whereas JA promotes overall leaf silicification and the maturation of phytolith-bearing silica cells by increase Si accumulation (Fauteux et al., 2006; Ye et al., 2013). During rice attacked by caterpillar *Cnaphalocrocis medinalis* (leaffolder, LF), significant decreases in Si deposition and an apparent loss of Si-induced LF resistance were observed in transgenic events that silenced the expression of either allene oxide synthase (OsAOS) or CORONATINE INSENSITIVE1 (OsCOI1), which is involved in JA biosynthesis or perception, suggesting that Si primes JA-mediated antiherbivore defense responses (Ye et al., 2013). Ubiquitin-protein ligase is suggested to be involved in the fine-tuning of JA-related response by degrading the JA-negative regulator, JAZ1 (Thines et al., 2007). Dreher and Callis (2007) demonstrated that up-regulation of ubiquitin-protein ligase by Si application in plants after pathogen infection may contribute to tuning the signaling of a defense response.

*JERF3, TSRF1* and *ACCO* are ET marker genes, *JERF3* is a transcription factor which is activated in response to ET and JA signaling, *ACCO* involved in ethylene biosynthesis, and *TSRF1* is an ET-responsive transcription factor (Pirrello et al., 2012). In tomato plants, the expression of *JERF3, TSRF1* and *ACCO* genes were upregulated by Si when challenging with *R. solanacearum*, supporting that Si induced resistance were mediated via ET and JA signaling pathways (Ghareeb et al., 2011). ET and JA interact to regulate the expression of particular defense-related genes such as *PDF1.2* upon pathogen perception (Pieterse et al., 2009) (**Figure 2**). In *Arabidopsis*, Si increased the *PDF1.2* expression during *Botrytis cinerea* infection, suggesting its role as a modulator of the signaling pathways involved in the plant’s response to fungal infection (Cabot et al., 2013). In rice*-C. miyabeanus* pathosystems, ET-insensitive *OsEIN2a* antisense plants were more resistance to brown spot than wildtype plants, and Si treatment of the *OsEIN2a* antisense transgenics or coapplication of Si and ET signaling blocker silver thiosulfate (STS) had no additive effect on brown spot resistance, suggesting that Si specifically targets the ET signaling pathway to defense resistance (Van et al., 2015a).

Three classes of active defense mechanisms are distinguished in plant–pathogen interactions regulated by Si application: the primary response comes in cells infected by pathogens; the secondary response is induced by elicitors and restricted to cells near to the initial infection site; and thirdly, the systemic acquired response is transported hormonally to all tissues of the infected plant (Hutcheson, 1998).

### Molecular Mechanisms

Silicon is involved in the metabolic processes of plant–pathogen interaction, activating defense genes of host plants via a series of physiological and biochemical reactions and signal transductions, as well as inducing the resistance response in plants to prevent plant diseases (Fauteux et al., 2005; Vivancos et al., 2015). Si may act in the primary response and modulate the activity of post-elicitation intracellular signaling systems which regulate the expression of defense genes related to structural modifications of cell walls, hypersensitivity responses, hormone synthesis, antimicrobial compound synthesis, and PR proteins (Fauteux et al., 2005).

**FIGURE 3:**
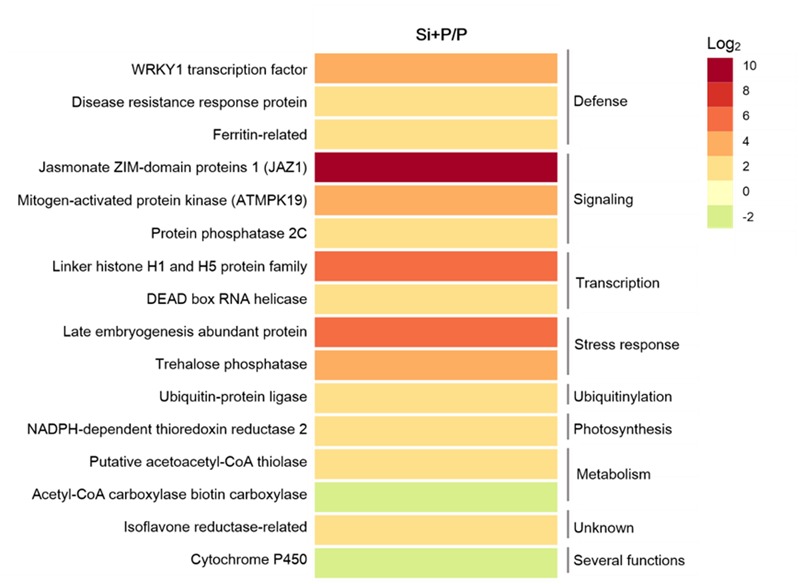
**Significantly regulated genes in response to silicon in tomato plants after infected with *Ralstonia solanacearum* for 72 h (Ghareeb et al., 2011).** The heatmap represents the ratio of genes expression between plants with silicon and inoculated with *R. solanacearum* (Si + P) and plants without silicon and inoculated with *R. solanacearum* (P).

Transcriptomic and proteomic studies have been conducted to illustrate the defense responses of Si in various pathosystems (Fauteux et al., 2006; Chain et al., 2009; Majeed Zargar et al., 2010; Ghareeb et al., 2011; Nwugo and Huerta, 2011). Si could induce tomato resistance to *Ralstonia solanacearum* via up-regulating the expression of genes involved in defense and stress responses, such as WRKY1 transcription factor, disease resistance response protein, ferritin, late embryogenesis abundant protein, and trehalose phosphatase (**Figure 3**) (Ghareeb et al., 2011). The similar result have been found in tomato stems of rhizobacteria and silicon treated-tomato genotypes upon inoculation with *R. solanacearum* compared to the non-treated, pathogen inoculated control, in which most of the up-regulated genes are involved in signal transduction, defense, protein synthesis and metabolism, while a large proportion of down regulated genes were involved in photosynthesis, lipid metabolism (Kurabachew et al., 2013). Crosstalk between signaling pathways in plant defense regulated by Si and related transcription factor have been detailed discussed in the Section of “Systemic Signals” and **Figure 2**. During the induction of systemic acquired resistance in cucumber mediated with Si, the expression of gene encoding a novel proline-rich protein (PRP1) was enhanced, which contributed to cell-wall reinforcement at the site of attempted penetration of fungi into epidermal cells (Kauss et al., 2003). During pathogen interactions in tomato plants (*R. solanacearum*), the expression of *CHI-II, GLU, PGIP*, and *POD*, which are attributed to virulence factors released by the pathogen to inhibit host resistance and facilitate host invasion, were down-regulated by Si application (Ghareeb et al., 2011). In tomato plants inoculated with *R. solanacearum*, 26 proteins were markedly changed by Si supply, suggesting that Si-mediated disease resistance may be related to change at a protein level (Chen et al., 2014).

Silicon could negate many transcriptional changes induced by pathogen infection, for example, *Arabidopsis* infected with the fungus *Erysiphe cichoracearum* results in alteration of the expression of a set of nearly 4000 genes, and the number or expression level of up-regulated genes, which are defense-related, were not changed compared with control and Si-treated plants, whereas the magnitude of the down-regulated genes, which are involved in primary metabolism, were attenuated when treated with Si (Fauteux et al., 2006). In wheat plants infected with *Blumeria graminis* f. sp. *tritici*, about 900 genes responding to pathogen infection were altered in control leaves, while few genes were changed by the pathogen in Si-supplied plants, suggesting that Si almost eliminated the stress imposed by the pathogen invasion (Chain et al., 2009). Similar findings were obtained by Brunings et al. (2009), the impact of *Magnaporthe oryzae* inoculation on the transcriptome of rice is diminished by Si application. Therefore, rather than inducing resistance by transcriptional reprogramming of defense-related genes, Si seems to eliminate the impact of pathogen infection on the transcriptome of host plants, probably through preventing the exploitation of pathogen virulence factors (Van et al., 2015b).

## Conclusion and Perspectives

**FIGURE 4:**
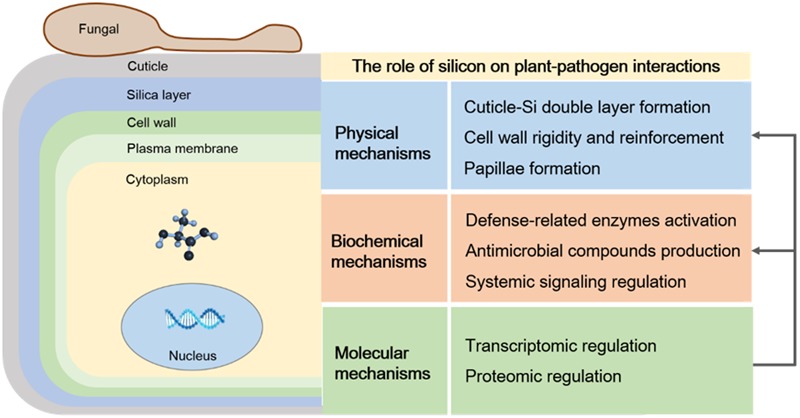
**The role of silicon (Si) on plant–pathogen interactions.** Si mediated plant defense responses were classified as physical, biochemical and molecular mechanisms. Physical mechanisms involved in cell wall reinforcement and papillae deposition, biochemical mechanisms were attributed to activating defense-related enzymes, stimulating antimicrobial compounds production as well as regulating the complex network of signals pathways, and the molecular mechanisms mainly contained the regulation of genes and protein related to defense responses.

By combining available information on the interaction of plant–microbes mediated by Si, the physical, biochemical, and molecular mechanisms that can be attributed to Si-mediated plant defense responses have been summarized in this review (**Figure 4**). Firstly, Si induces resistance against a wide range of diseases by acting as a physical barrier, which is based on pre-formed defense barriers before pathogen infection, for example, wax, cuticle, and cell-wall protection, and post-formed defense barriers after pathogen infection, for example, cell-wall reinforcement and papillae deposition at infection sites. Secondly, Si-induced biochemical resistance during plant–pathogen interactions involves activating defense-related enzymes activates, stimulating antimicrobial compound production, and regulating the complex network of signal pathways. Finally, Si may act at a molecular level to regulate the expression of genes involved in the defense response. Understanding plant–microbe interactions regulated by Si will be helpful in the effective use of this mineral to increase crop yield and enhance resistance to plant diseases. Although numerous studies have elucidated the possible mechanism of Si-mediated resistance at the physical, biochemical, and molecular levels, detailed mechanisms of Si regulated plant–microbe interactions, such as plant signaling transduction and transcriptome regulation of defense-related pathways, are needed for further study.

## Author Contributions

MW and SG wrote the manuscript; LG contributed in the tables; SD and YS contributed in the figures; QS and SG revised the manuscript.

## Conflict of Interest Statement

The authors declare that the research was conducted in the absence of any commercial or financial relationships that could be construed as a potential conflict of interest.
